# Vertical head rotation as major source of differences between time-separated digital cephalometric radiographs of patients acquired in one cephalostat X-ray device

**DOI:** 10.1186/s12880-022-00935-x

**Published:** 2022-11-24

**Authors:** Ralf K. W. Schulze, Lea K. M. Linnerth

**Affiliations:** 1 Devision of Oral Diagnostic Sciences, Departmet of Oral Surgery and Stomatology, University of Bern, Freiburgstr. 7, 3010 Bern, Switzerland; 2 Private Dental Office, Theodor-Heuss-Str. 22, 55239 Gau-Odernheim, Germany

**Keywords:** Cephalometric radiography, Head position, Repeated assessments, Cephalometry

## Abstract

**Background:**

The purpose of the retrospective study was to analyze the reliability and repeatability of specific landmark-positions used in cephalometry to determine the major sources of absolute landmark position differences for repeated, time-separated (time-point I and II) digital cephalometric radiographs (CEPH) of the same patients.

**Methods:**

100 pairs of CPEHs from the database of a sample of adult patients (18 to 28 years) were analyzed by one calibrated observer and three landmark points (Sella: S, Nasion: N, Subspinale: A) were digitally marked using ImageJ-software. The coordinates of these points entered the evaluation using displacement vectors as primary endpoints between the coordinates of the landmarks in the two images as well as SNA-angles and the angle ω of SN relative to the floor.

**Results:**

Displacement vectors between CEPHI and CEPHII were rather large (N: 7.95 ± 4.85 mm, S: 5.34 ± 3.50 mm, A: 4.81 ± 3.95 mm. SNA was rather stable between the two sequential radiographs (mean difference: 0.002° ± 1.85°). and did not correlate with age of the patient (SNA_I_: spearman-Rho: 0.0239, *p* = 0.8134; SNA_II_ : spearman-Rho: 0.0244, *p* = 0.8096). Although the vertical angle ω did not differ between CEPH_I_ and CEPH_II_ (mean difference: 0.4° ± 4.7°, p_wilcoxon_ = 0.8155), it showed a quadratic relationship (p_F−statistic_: < 2.2e−16) with the length of the displacement vector N.

**Conclusion:**

The significantly varying location of the reference points S, N and A between time-separated CEPHs of one patient can largely be explained by different angulation (head rotation within the sagittal plane) of the Frankfurt plane to the floor (horizontal plane).

## Background

Cephalometry on lateral cephalometric radiographs (CEPH) represents the radiographic backbone in orthodontic planning treatment [1, 2]. Commonly, CEPHs are repeatedly acquired to monitor growth of a patient over the entire treatment [1].

A cephalometric radiograph applies an effectively standardized imaging geometry using paralleling technique, i.e. the median-sagittal plane is orientated parallel to the detector plane [3]. In combination with a relatively large source-to-detector-distance (generally > 1.5 m) the resulting projection radiograph can be metrically assessed within certain narrow error margins. In this context we notice that regardless of a standardized projection geometry a projection radiograph inherently reduces 3D space into a flat 2D image, i.e. mathematically reduces the dimensions from 3 to 2 [4]. Despite this inherent shortcoming affecting all projection radiographs, CEPHs are used routinely for the purpose of sagittal and vertical dimensional maxillofacial structures assessment [5]. The efficacy of cephalometric imaging in orthodontic treatment, despite its long-term world-wide application is still under debate. Nijkamp and colleagues concluded from their results that “that cephalometrics are not required for orthodontic treatment planning, as they did not influence treatment decisions” [6]. Along with this ongoing debate, many investigations on factors influencing the accuracy of cephalometric image analysis have been published [7–10]. It has been concluded that predominantly differences in head-position, particularly head-rotation about the vertical patient axis introduces errors in the images [11]. As typically cephalostats place ear-rods bilaterally into the patients’ meatus accusticus externus yet the glabella-rest may be vertically adapted, a head rotation within the sagittal plane may easily occur if the horizontal reference plane is not sufficiently orientated. Generally, CEPHS either use the sella-nasion-plane (S–N-plane) or the Frankfort-plane as horizontal reference plane [12]. Obviously, the former cannot readily be applied for CEPH-acquisition since sella is a constructed point located in the base of the skull and thus is not visible from the outside. Hence, commonly the Frankfort plane is used as reference. Repeatability of landmark identification on CEPHs is critical for clinical decision making and treatment monitoring. Lagavere and colleagues estimate that mean differences in cephalometric landmark identification less than 1 mm are clinically acceptable and those between 1 and 2 mm are still usefull for most analyses [13]. Similar clinical accuracy was postulated in the 1980ies already [14]. To assess facial growth, it is essential that the cephalometric analysis can clearly discriminate between differences caused by growth and those caused by other factors. The latter is mainly relating repeatability of cephalometric landmarks which has been investigated by many authors before (see, e.g [13, 15, 16]). Despite this rather large body of literature, we did not find a study comparing the repeatability of certain landmarks in CEPHs of one patient between different time-instances (I,II). In other words, if a patient is positioned twice or several times in the same X-ray unit (cephalostat), how reproducible are the absolute positions of landmarks as well as their positions to one another in the resulting images? Considering the fact that CEPHS are among the most-standardized two-dimensional projection radiographs, one interesting question is the absolute repeatability achieved by this standardization. Hence, the aim of this retrospective study was to analyze the major sources of absolute differences in position of typical landmarks used for cephalometric evaluation. In using repeated time-discriminated CEPHs of clinical patients and evaluating the absolute geometrical positions of some distinct landmarks we aim to clarify which parameters mainly contribute to changes in landmark position between images. Given no changes in the facial skull and the soft tissue and a correctly controlled horizontal orientation, one would expect that in an ideal situation, in which the patient was positioned absolutely identically in the X-ray unit, the reference/landmark points would be depicted at identical locations in the resulting CEPHs. Hence the null hypothesis was that the locus of depiction is the same in CEPH_I_ (acquired at time point I) and CEPH_II_ (acquired at time point II).

## Methods

100 pairs of patient CEPHs taken from the database of the Dental School at the University Medical Center of Mainz, Germany acquired between 01.01.2015 and 01.08.2018 entered the retrospective analysis. No radiograph was acquired for this study. Requirement were that patients were over 18 years old to exclude facial growth effects. CEPHs were processed in a pseudonymized fashion (only age and sex were recorded). Patients at the University Medical Center of Mainz sign a general consent that anonymized records and images may be used for scientific purposes if the data cannot be used for later identification. As no further information was used and no patient data apart from those processed pseudonymized data are included in the manuscript, ethical approval is not required according to the regulations at University Medical Center of Mainz (Ethics committee of the Rhineland-Palatinate Chamber of Physicians). If more than two CEPHs were available for a patient, those two with the smallest time-interval in between were selected for the analysis. Inclusion criteria were:

- 2 CEPHs from the same patient acquired at two separated time-instances (time point I and II).
- CEPHs were all acquired with one digital cephalometric radiographic unit (Orthophos XG plus DS/Ceph (Sirona Dental Systems GmbH, Bensheim, Germany; specifications see Table 1).
- Minimum patient age at the time of image acquisition: 18 years under the assumption that the main facial growth effects in the area of the landmarks investigated in this study should be terminated at that age.
- No surgical intervention or facial trauma between first and second image. However, conservative orthodontic treatment between the two time-points was allowed.

**Table 1:** Technical characteristics of the digital cephalometric X-ray unit

Parameter	Specification
Active area of detector	CCD-detector, 230 mm × 6.48 mm
Physical pixel size	0.027 mm
Processed pixel size (4 × 4 binning)	0.104 mm
Source-to-detector-distance	1714 mm
Source-to-object-distance (object: median-sagittal plane of patient)	1500 mm
Magnification factor in median-sagittal plane	1.14
Exposition time (slot technique)	4.7 to 9.4 s
Focal spot size	0.5 (IEC 336)

Our staff consisting of three radiographic technicians and two dental assistants is generally instructed to carefully position the patients using the ear-rods plus the glabella-rest of the cephalostat integrated in the digital panoramic X-ray machine. All CEPHs were taken using the cephalostat integrated in the X-ray machine (nasal support plus ear rods) with the Frankfort-plane orientated parallel to the floor. This position is in accordance with generally agreed criteria [17]. The X-ray device applies a horizontal scanning slot-technique to acquire the CEPH by means of a vertically orientated CCD-line-sensor (see Table 1).

Technical data of the X-ray unit is presented in Table 1. According to the radiation protection standards at the Section of Oral and Maxillofacial Radiology, University Medical Center of Mainz, exposures of the CEPHs had been collimated to the required area.

### Evaluation of the digital CEPHs

Images were exported from the X-ray unit into „ImageJ “ (National Institutes of Health, Maryland, USA; https://imagej.nih.gov/ij/download.html) as uncompressed tiff-format. To avoid additional sources of error we only selected three stable landmark (reference) points fulfilling the following criteria:

- Position within the mid-sagittal plane.
- Common application of these selected landmark points.
- Landmark points outside mandible to avoid addition positioning ambiguities due to mandibular position relative to remaining facial skull.

**Fig. 1:**
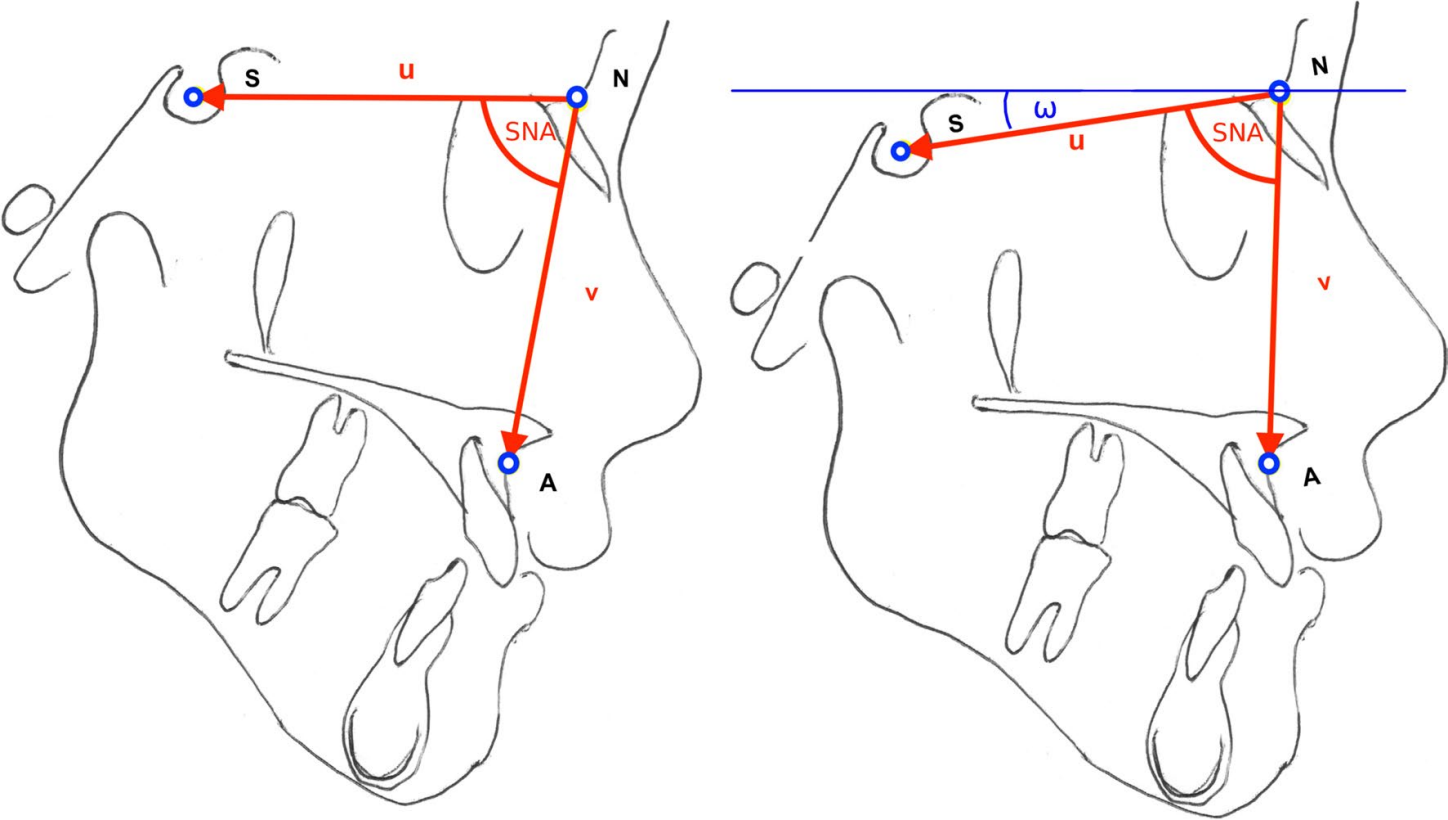
Sketch of the reference points S, N and A, the SNA-angle and the horizontal angle ω. For illustration purposes, here SN in the left image is ideally orientated parallel to the horizontal plane, hence the ω = 0° in this case

These criteria suggested the following three landmark points (Fig. 1):

1. Sella “S”: geometric midpoint within the bony contour of the sella turcica.
2. Nasion “N”: the most anterior point of the frontonasal suture in the midsagittal plane.
3. Subspinale “A”: most caudally located point deepest point of the curvature of the surface of the maxillary bone between ANS and the alveolar crest of the maxillary central incisor (according to Downs [18]).

Using ImageJ, these landmark points were marked by one observer (XX) on an Eizo mx215 radiforce 21.3 inch LCD-monitor in 1:1 display, i.e. each (binned) image pixel is displayed in one monitor pixel. The reference points were marked with the mouse-driven cursor in each image-pair and the x-/y-pixel-coordinates of each point exported from ImageJ into a spread-sheet software (Microsoft^®^ Excel 2017, Redmond, WA, USA).

### Statistical data evaluation

Image coordinates of each of the three landmarks were exported from Microsoft Excel as comma-separated values (*.csv) and further processed in R language and environment for statistical computing (R Foundation for Statistical Computing, Vienna, Austria [19]. The level of significance was set at 5%. To compute absolute distances pixels were multiplied with pixel-size (Table 1). Data are reported with two digits as the originally exported coordinates were also given with two digits. To evaluate differences in imaging-position, one common coordinate system was established. Following a typical convention in computer science, we established the top left corner pixel of the CEPHs as origin (0,0). Accordingly, pixels from there are counted from left to right and top to bottom. As all images were acquired with the same (identical) X-ray machine, this coordinate system ensured that all differences in coordinates represent differences in location of the reference point producing structures during exposure. The device continuously since its installation underwent monthly obligatory consistency tests as required by the German national standard (DIN 6868-5:2020-05), which also controls for geometric accuracy of the machine. Vectors using the x and y-coordinates were computed for all reference points equally, e.g. for point A:

1 $$\begin{aligned} A_{x} & = \, \left| {A_{XI} } \right| - \left| {A_{XII} } \right| \\ A_{y} & = \, \left| {A_{yI} } \right| - \left| {A_{yII} } \right| \\ \end{aligned}$$

and the respective distance |**A|** (length of vector A) between position I and II then computed from:

2 $$\left| A \right| = \sqrt {A_{x}^{2} + A_{y}^{2} }$$

The longer |**A|**, the more the location/position of this reference point deviates between the two images CEPH_I_ and CEPH_II_. Vectors $u$ and $v$ (see Fig. 1) were computed analogously.

In addition, the angle SNA (Fig. 1) was calculated from the vectors $u$ and $v$ from the dot product (*) as follows:

3 $$cos\varphi = \frac{u*v}{{\left| u \right| \cdot \left| v \right|}}$$

For all 100 image-pairs, differences in this angle (in degrees) as well as length-differences between CEPH_I_ and CEPH_II_ were computed per patient as primary endpoint variables.

To evaluate the horizontal orientation of the patient the angle ω between vector S–N (computed analogously using Eqs. 1 and 3) and the horizontal plane was computed for each CEPH and compared between CEPH I and II. For definition of the horizontal plane, an auxillary vector *h* was constructed parallel to the pixel-row through the y-coordinate of S and an auxillary point H (x_N_.y_S_). Vectors $u$ and $h$ encompass the angle ω defining the horizontal angulation of the patient during the exposure. Spearman correlation was used to evaluate linear relationships while a quadratic regression model was used to explain the variance in the data between ω and the vector lengths.

Repeatability in landmark identification was assessed as intra-observer-variation for a subset of 20 CEPHs. For this purpose, the initial 20 CEPHs as retrieved from the database were selected. These were assessed twice by one observer (XX) on the non-annotated CEPHs with a minimum time-interval between assessments of 30 days to avoid a potential memory bias. Bland–Altman-Plots [20] were produced separately for x- and y-coordinates to assess intra-observer repeatability.

## Results

Age of the patient sample (71 females and 29 males) ranged between 18 and 78 years (mean, ± standard deviation: 35 ± 13.8 years). Mean time difference (time interval) between the two radiographs was 2.11 ± 1.53 years. (range: 0.08–11.92 years.).

**Fig. 2:**
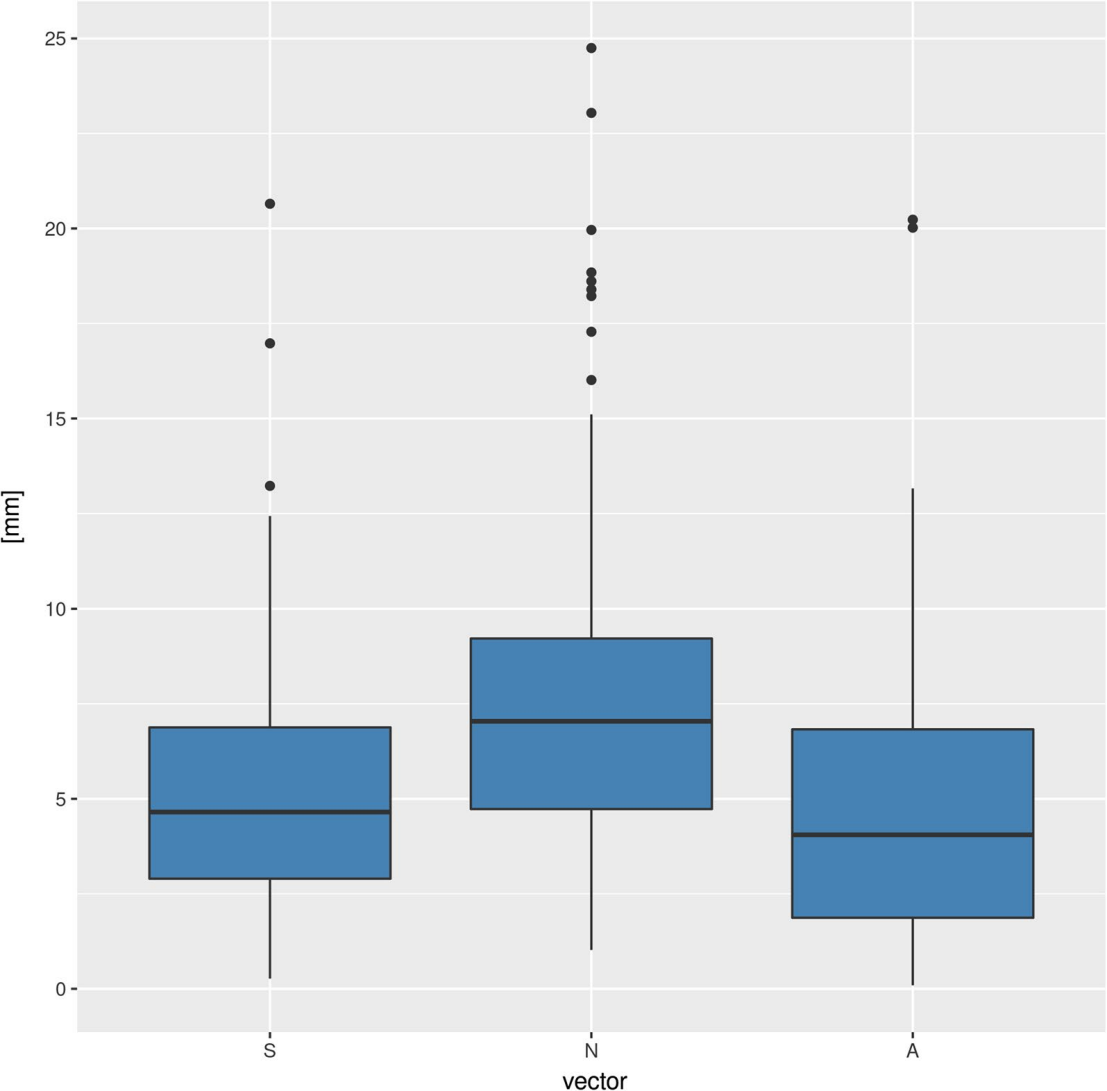
Length of displacement vectors A, N and S describing differences in location between the reference points between the two radiographs CEPH_I_ and CEPCH_II_

The largest deviations as represented by the length of the vector were found for nasion (N) (mean ± standard deviation: 7.95 ± 4.85 mm, median: 7.04 mm), followed by S (mean ± standard deviation: 5.34 ± 3.50 mm, median: 4.65 mm) and A (mean ± standard deviation: 4.81 ± 3.95 mm, median: 4.05 mm, Fig. 2). None of the deviation vectors significantly correlated with age.

**Fig. 3:**
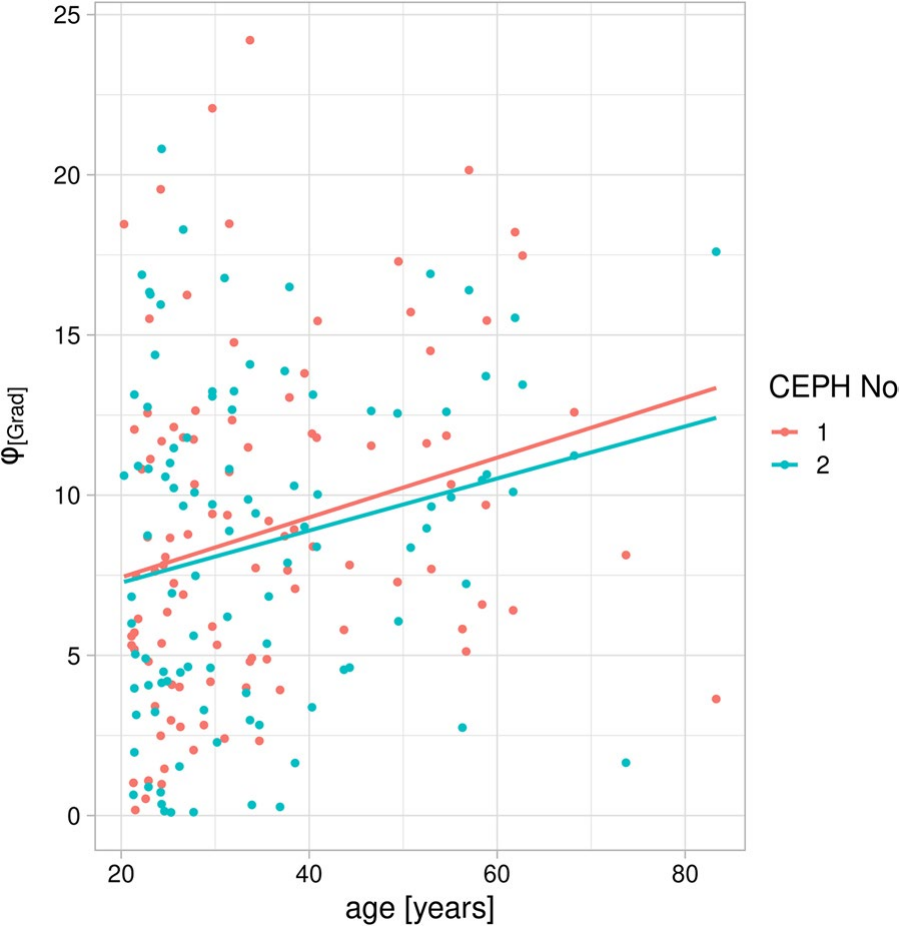
In both CEPHs there was a significant positive correlation between the horizontal angulation ω during image acquisition and age of the patient

The vertical angulation of SN relative to the horizontal plane (mean CEPH_I_: 8.8° ± 5.2°, mean CEPH_II_: 8.5° ± 5.1°) as expressed by the angle ω did not differ between CEPH_I_ and CEPH_II_ (p_wilcoxon = 0.8155) and slightly positively correlated with age (Fig. 3: CEPH_I_: spearman Rho = 0.311, *p* = 0.0016; CEPH_II_: spearman Rho = 0.182, *p* = 0.0695). Mean difference for this angle was 0.4° ± 4.7°, yet we observed a rather large range from − 14.4° up to 11.2°.

**Fig. 4:**
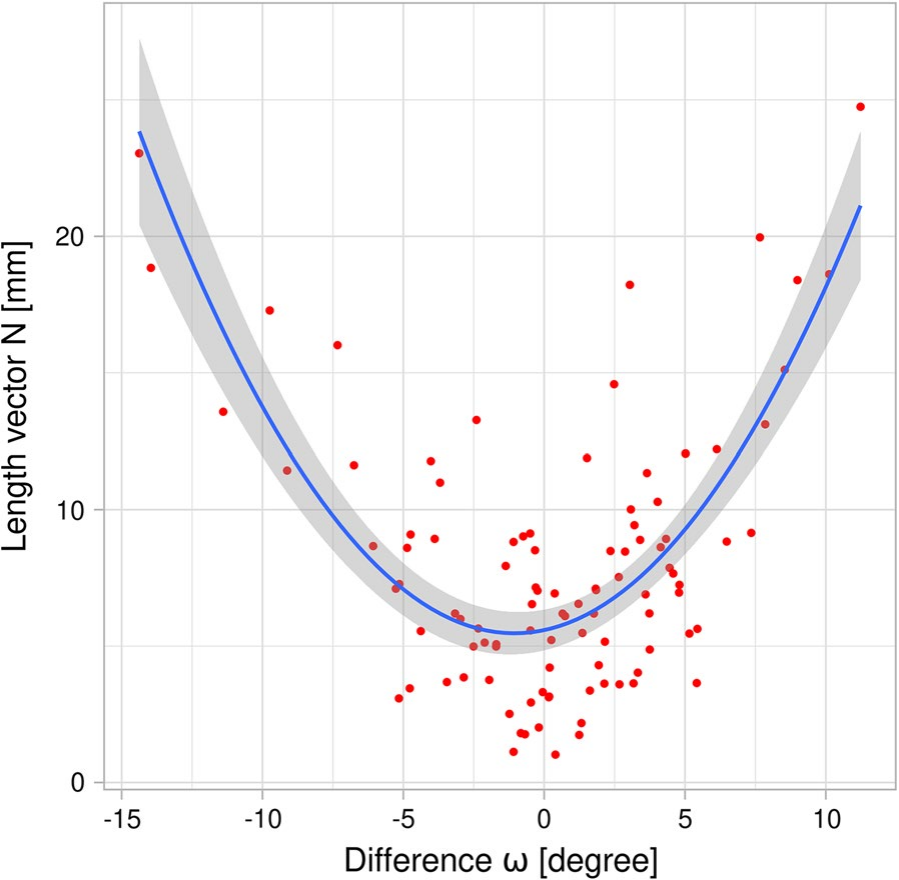
Displacement-vector N between CEPH_I_ and CEPCH_II_ exhibits a quadratic relationship with ω: if the latter was close to zero, N also reached minimum length

Interestingly, the difference between ω_I_ and ω_II_ exhibited a quadratic relationship with the length of the error vector N. 68.35% of the variation in the data are explained by this model (Fig. 4, p_F-statistic_: < 2.2e–16). In other words, the the larger the vertical angulations (ω) differences in both directions, the more the location of point N also varied between the sequential CEPHs.

No correlation was found for differences in SNA between the two CEPHs and age of the patient (R_Spearman_ =  − 0.0167, *p* = 0.8687). Neither was a significant correlation observed between absolute differences in SNA and time-difference between the two CEPHs (R_Spearman_ =  − 0.0550, *p* = 0.5865). SNA despite some few outliers (range: − 5.33°; 11.15°) was stable between the two sequential radiographs (mean difference: 0.002° ± 1.85°). Neither correlated SNA for any of the two images with age of the patient (SNA_I_: spearman Rho: 0.0239, *p* = 0.8134; SNA_II_: spearman Rho: 0.0244, *p* = 0.8096).

Intra-rater repeatability was excellent, with mean differences (± standard deviation) of entirely below 1 mm (Table 2). The vertical direction (y-coordinate) was less reproducible than the horizontal one (x-coordinate). All differences (except the x-coordinate of S) followed a normal distribution, hence the assumptions on the limits of agreement are valid.

**Table 2:** Intra-rater-reproducibility of landmark definition assessed for 20 cases

Reference point	Coordinate	Mean difference [mm]	Standard deviation [mm]	Range [mm]
S	x	–0.012	0.091	–0.150; 0.260
S	y	0.004	0.114	–0.310; 0.160
N	x	0.026	0.119	–0.150; 0.310
N	y	0.118	0.189	–0.310; 0.430
A	x	–0.075	0.110	–0.290; 0.170
A	y	0.081	0.261	–0.310; 0.540

Differences were defined as measurement No1 minus measurement No2

## Discussion

Repeated cephalometric radiography is commonly used as fundamental tool for monitoring orthodontic treatment outcome over months or years [1, 21, 22]. Obviously, this also applies for digital CEPHs which represent the current standard. Interestingly, however, a systematic review from 2013 concluded that “scientific evidence on the usefulness of this radiographic technique in orthodontics is still lacking, with contradictory results” [5]. While our study due to its design cannot provide conclusion on accuracy of cephalometric assessments, however, it provides some insight into the repeatability of landmark definition in repeated (sequential) CEPHs acquired in a single digital machine. From the technique itself and its highly standardized imaging geometry it should be expected that two time-separated CEPHs of the same patient in the same X-ray unit should produce rather similar results. Yet our observations suggest a relatively low level of agreement between two CEPHs, with maximum deviations of ca. 25 mm for Nasion (mean 7 mm, Fig. 2). Of course this is an outlier yet still a deviation that occurred in this study. For sella and subspinale these values are somewhat smaller, yet still in the range of several millimeters (Fig. 2). It is important to provide absolute values here and not only correlation results to obtain an impression on clinical relevance of the values. We consider so many millimeter a relevant deviation, yet what does that mean in the context of our research? First of all it represents a difference between two CEPHs acquired at two time-instances separated by several months or even years. The mean was somewhat over 2 years, yet we also had some separated by more than ten years. Although we excluded patients younger than 18 years when the first CEPH was taken, most probably some bony morphological changes also occur in an adult patient population that was on average 35 years old. Although orthodontic treatment apart from surgical interventions was also allowed to happen between the two CEPHs, the selection of maxillofacial landmark points ensured that these won’t be much affected by such treatment to the dentition. However, since point A is primarily alveolar this point may have been affected by otthodontic treatment in between the two time points. While contradicting statements regarding SNA-changes with age have been reported, [23–25] we found no correlation with age within our sample. Also, contrary to what was observed by Bondevik and colleagues [25], SN did not change with age. However, it should be mentioned here that these potential changes with age were not subject of this study. Rather these results should interpreted as indicator in how far age-related effects explain differences between CEPHs in this study. From our observations we may conclude, that age as represented in our sample was not a real influencing factor on the outcome.

It has been long known that definition of cephalometric reference points involve systematic errors, [26] the dimension of which is different between different landmarks [21, 27]. Even the intra-observer error for repeated assessment of the same CEPH seems to significantly differ between different landmarks [28].

Both nasion as well as subspinale represent landmarks in the midline and located in curvatures. Landmarks in curvatures are known to be more error-prone than those located on edges [5]. We nevertheless selected those landmarks to assess commonly utilized landmarks in the sense of a “representative” sample of reference points, all located in the mid-sagittal plane.

One drawback of our study design is the involvement of only one observer (LL) thus not considering a well-known inter-observer variance [28]. To at least obtain data on the repeatability of the measurements in this single observer, we conducted two separate evaluations for a subsample of 20 patients. To avoid learning effects, the landmark points were marked in two sessions separated by 30 days. It turned out the mean intra-rater variation as expressed by Euclidean distances was entirely below one millimeter for all three reference points (Table 2). This exceptional intra-observer agreement may be explained by the fact, that regardless of the 30 days interval, the training effect from the first evaluation round, resulted in a high accuracy in manual landmark definition.

**Fig. 5:**
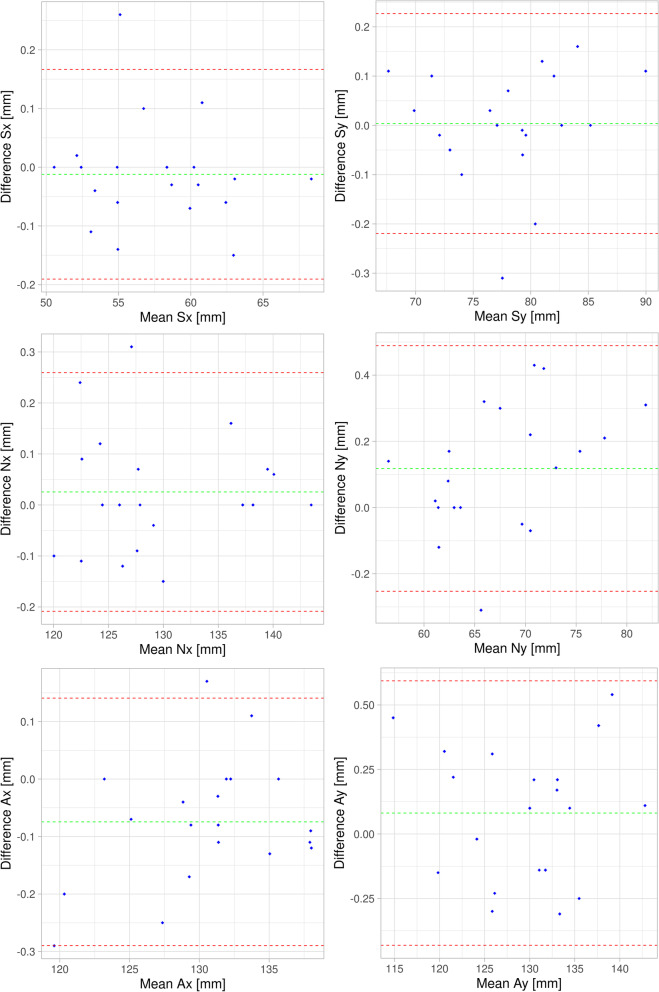
Bland–Altman plots [20] illustrating intra-observer variation assessed for the subset of 20 CEPHs. For each point and coordinate (x,y), differences between assessment No 1 and 2 (x-axes) are plotted against the mean of both assessments. The limits of agreement are displayed as mean (green dashed line) ± 1.96 standard deviation (dashed red lines)

Our observations strongly suggest that the major source of differences observed in our study is due to head-rotation within the sagittal plane. This hypothesis is supported by several factors. First, the orientation to the horizontal plane as indicated by the angle ω between SN and the horizontal image plane varied considerably more between the two images (range of differences ω: − 14.38°, 11.23°, mean: 0.35°) than SNA (range of differences SNA: − 5.33°, 11.15°, mean: − 0.0023°). Second, it turned out that the length of vector N, i.e. the one that showed the largest differences between the two images indicated a highly significant quadratic relation with the difference between ω_I_ and ω_II_. In other words, if the horizontal angulation of the patient during exposure (expressed by ω) was similar for CEPH_I_ and CEPH_II_ (i.e. the difference between ω_I_ and ω_II_ close to 0), N was also smallest (Fig. 5). As in a cephalostat the center-axis of potential rotation is defined by the ear-rods inserted into the external acoustic meatus, it is also likely that N being furthest away from this rotation axis undergoes the largest rotation-distance between two separate exposures. However, this argumentation is somehow contradicted by the observation that S being closest to the rotation axis, in our sample showed the second largest deviation. Although our staff in the Section of Oral Radiology was instructed to adjust the Frankfort plane properly for every single CEPH and despite the fact, that ca. 1000 of such radiographs are being acquired there per year, correct orientation within the vertical plane seems challenging and prone to significant variation. We did not investigate the effects of such rotation on linear and angular measurements as e.g. in [9]. From the small variation we observed for SNA, however, it is obvious that, as expected, this angle is not significantly affected by rotation occurring in the sagittal plane. This follows from the simple logic that an angle is invariant to rotations occurring within its plane. As our landmarks are all located in the mid-sagittal plane of the patient which is parallel to the imaging-plane, this assumption will hold true.

From a clinical perspective it can be concluded, that the major differences we observed between two CEPHs of the same patient taken at two time-points are explained by head rotation within the sagittal plane. While these will not significantly affect linear and angular measurements, they will hamper a direct comparison between the images. For the latter, the images require rotation within this plane (i.e. the image plane) to be registered to one another. While this can be certainly done automatically using either conventional image registration or artificial neuronal networks, the angular differences will surely affect the soft-tissue visualization. Since these are affected by gravity, it may be speculated that the soft-tissue profile may significantly differ between the two CEPHs if also ω differs considerably. Yet Hoogeveen and coworkers had observed, that such effects are only minimal for the facial soft-tissue profile. [29] As a potential hardware solution, we believe the significant differences in vertical head angulations between sequential cephalometric radiographs of the same patient could possibly be minimized by stringent use of a device-integrated laser beam indicating the desired orientation of the Frankfort plane.

## Conclusions

In conclusion, we observed significant differences in the location of the reference points S, N and A between time-separated CEPHs of one patient. The major source of these differences, however, can be explained by different angulation (head rotation within the sagittal plane) of the Frankfurt plane to the floor (horizontal plane).

## Data Availability

The datasets used and/or analysed during the current study are available from the corresponding author upon reasonable request.

## Abbreviations

CPEH: Cephalometric radiograph
2D: Two-dimensional
3D: Three-dimensional
CCD: Charged-coupled-device
LCD: Liquid crystal display
S: Reference point "Sella"
N: Reference point "Nasion"
A: Reference point "Subspinale"
SNA: Angle spanned between the reference points SNA
